# Supplementary material for: Deciphering novel common gene signatures for rheumatoid arthritis and systemic lupus erythematosus by integrative analysis of transcriptomic profiles
Source: PLoS One. 2023 Mar 16;18(3):e0281637. doi: 10.1371/journal.pone.0281637 (PMC10019710; doi:10.1371/journal.pone.0281637)
Supplement: S3 Table — (DOCX) [file pone.0281637.s003.docx]

| Gene name | Effectsize | nstudies | Gene name | Effectsize | nstudies |
| --- | --- | --- | --- | --- | --- |
| ANXA3 | 0.822525971 | 7 | ABCA1 | 0.442511271 | 7 |
| CAMP | 0.772005697 | 7 | CYP1B1 | 0.440098719 | 7 |
| TNFAIP6 | 0.723657312 | 7 | ICOS | 0.433879415 | 7 |
| PHTF1 | 0.718647311 | 7 | PGM3 | 0.430397549 | 7 |
| ACVR2A | 0.709280042 | 7 | PLAUR | 0.42037731 | 7 |
| COL17A1 | 0.62085862 | 7 | B4GALT5 | 0.418552469 | 7 |
| CLDND1 | 0.609250385 | 7 | CD226 | 0.41610023 | 7 |
| SLC22A4 | 0.601572511 | 7 | EDA | 0.410633243 | 7 |
| SRGN | 0.589594132 | 7 | ADAMDEC1 | 0.409295019 | 7 |
| HMGB2 | 0.577371867 | 7 | OASL | 0.405973339 | 7 |
| LHFPL2 | 0.545276617 | 7 | MLF1 | 0.40385119 | 7 |
| DEFA4 | 0.540173033 | 7 | BPGM | 0.400811453 | 7 |
| PTGS2 | 0.532689537 | 7 | SFT2D1 | 0.798199681 | 6 |
| NFIL3 | 0.523637677 | 7 | TNFSF13B | 0.761034699 | 6 |
| PLOD2 | 0.517568516 | 7 | ATP8B4 | 0.74832098 | 6 |
| RGS2 | 0.484633397 | 7 | LCOR | 0.73043524 | 6 |
| FAM76A | 0.477060116 | 7 | SNX16 | 0.726171746 | 6 |
| SMURF2 | 0.475346834 | 7 | ZBTB41 | 0.691680086 | 6 |
| SACS | 0.461641158 | 7 | ZNF292 | 0.643582385 | 6 |
| ARG2 | 0.457833143 | 7 | SLC26A8 | 0.636056406 | 6 |
| B2M | 0.456159117 | 7 | CENPQ | 0.626284129 | 6 |
| CCNA2 | 0.455750226 | 7 | CXCL1 | 0.625702921 | 6 |
| CD200 | 0.45397512 | 7 | TBC1D19 | 0.602026886 | 6 |
| MLH3 | 0.453420158 | 7 | EFCAB2 | 0.575947373 | 6 |
| CTNNAL1 | 0.452869402 | 7 | RGAG1 | 0.575644456 | 6 |

**RA top 50 up-regulated genes**

**RA top 50 down-regulated genes**

| Gene name | Effectsize | nstudies | Gene name | Effectsize | nstudies |
| --- | --- | --- | --- | --- | --- |
| ZMYM3 | -0.40039113 | 7 | POLR3C | -0.524056474 | 7 |
| ANAPC5 | -0.40630923 | 7 | BCAT2 | -0.527067343 | 7 |
| USP13 | -0.408076457 | 7 | BCS1L | -0.527538902 | 7 |
| CBX7 | -0.408857983 | 7 | VARS | -0.527963584 | 7 |
| IL3RA | -0.410791221 | 7 | MAPK3 | -0.528226917 | 7 |
| PDAP1 | -0.416507742 | 7 | DNAJB1 | -0.534089428 | 7 |
| DDX42 | -0.417662797 | 7 | EIF4B | -0.53424793 | 7 |
| CTNNBIP1 | -0.420241685 | 7 | ARL2BP | -0.539870611 | 7 |
| SSSCA1 | -0.425046605 | 7 | DNAJB2 | -0.543600486 | 7 |
| MAN2B2 | -0.43321118 | 7 | ALDOC | -0.546658025 | 7 |
| VEGFB | -0.436833473 | 7 | KLHL25 | -0.547159894 | 7 |
| RBM4 | -0.443674646 | 7 | RPL28 | -0.548396575 | 7 |
| LDOC1 | -0.459418035 | 7 | EIF4EBP3 | -0.549543167 | 7 |
| POLG2 | -0.462825481 | 7 | G3BP1 | -0.553180313 | 7 |
| EWSR1 | -0.464182015 | 7 | HIVEP2 | -0.553931641 | 7 |
| MAPRE1 | -0.476053704 | 7 | TGFBRAP1 | -0.568283545 | 7 |
| SCARB1 | -0.476528321 | 7 | ABHD14A | -0.568796718 | 7 |
| DDX10 | -0.482429667 | 7 | SAMM50 | -0.577171067 | 7 |
| TATDN2 | -0.484242598 | 7 | SLC10A3 | -0.579547188 | 7 |
| DDX19B | -0.493156666 | 7 | RALY | -0.582000214 | 7 |
| TSR1 | -0.499601315 | 7 | ATP5D | -0.587722748 | 7 |
| LAG3 | -0.507118786 | 7 | ACO1 | -0.590595045 | 7 |
| TELO2 | -0.515728737 | 7 | NDUFS8 | -0.602723528 | 7 |
| KCNA3 | -0.52187039 | 7 | TGIF2 | -0.608595246 | 7 |
| AHCY | -0.522417742 | 7 | FOXJ3 | -0.611997319 | 7 |

**SLE top 50 up-regulated genes**

| Gene name | Effectsize | nstudies | Gene name | Effectsize | nstudies |
| --- | --- | --- | --- | --- | --- |
| IFI44L | 2.469662435 | 7 | SIGLEC1 | 1.437492526 | 7 |
| HERC5 | 2.286601502 | 7 | RTP4 | 1.435577628 | 7 |
| IFIT3 | 2.249519577 | 7 | CHMP5 | 1.42297887 | 7 |
| ISG15 | 2.202706499 | 7 | IFIH1 | 1.408675568 | 7 |
| IFI27 | 2.128954502 | 7 | LAP3 | 1.405740901 | 7 |
| RSAD2 | 2.094387057 | 7 | IFIT2 | 1.399675836 | 7 |
| IFI44 | 2.036250741 | 7 | IFI16 | 1.399489644 | 7 |
| IFIT1 | 2.011622526 | 7 | CLIC1 | 1.395807021 | 7 |
| MX1 | 1.948010018 | 7 | NMI | 1.36940084 | 7 |
| OASL | 1.889199094 | 7 | HERC6 | 1.359646583 | 7 |
| MX2 | 1.859692975 | 7 | SQRDL | 1.358321748 | 7 |
| PLSCR1 | 1.776554199 | 7 | PARP12 | 1.347188647 | 7 |
| IFITM3 | 1.769471992 | 7 | TNFAIP6 | 1.336283459 | 7 |
| TDRD7 | 1.703978668 | 7 | LAMP3 | 1.332858808 | 7 |
| OAS1 | 1.648717198 | 7 | IFI6 | 1.324685795 | 7 |
| OAS2 | 1.624937177 | 7 | IFIT5 | 1.307674224 | 7 |
| TAP1 | 1.602884436 | 7 | SP110 | 1.305671569 | 7 |
| SAMD9 | 1.598328626 | 7 | TNFSF10 | 1.293183913 | 7 |
| IFI35 | 1.509779242 | 7 | ISG20 | 1.29258158 | 7 |
| LY6E | 1.508281541 | 7 | ZBP1 | 1.257767441 | 7 |
| SCO2 | 1.492167454 | 7 | STAT1 | 1.233698011 | 7 |
| OAS3 | 1.455174897 | 7 | TOR1B | 1.202590983 | 7 |
| IRF7 | 1.447958325 | 7 | ANXA3 | 1.202492528 | 7 |
| IFITM1 | 1.447112013 | 7 | PHF11 | 1.201744123 | 7 |
| BST2 | 1.438450112 | 7 | SERPING1 | 1.167458394 | 7 |

**SLE top 50 down-regulated genes**

| Gene name | Effectsize | nstudies | Gene name | Effectsize | nstudies |
| --- | --- | --- | --- | --- | --- |
| PARD6B | -0.400375911 | 7 | KRT37 | -0.422298919 | 7 |
| AKAP1 | -0.40077152 | 7 | ITM2A | -0.422790687 | 7 |
| TNFSF11 | -0.401541215 | 7 | NDUFA5 | -0.422816929 | 7 |
| CAMSAP1 | -0.402602709 | 7 | CBL | -0.422826031 | 7 |
| DMWD | -0.404362337 | 7 | IDH3B | -0.423003627 | 7 |
| AKR1C3 | -0.404911294 | 7 | NFYB | -0.423273637 | 7 |
| RPL24 | -0.406262769 | 7 | SLC27A5 | -0.424299346 | 7 |
| MAPK8 | -0.406974289 | 7 | ITGB1BP2 | -0.424845712 | 7 |
| DHX30 | -0.407380897 | 7 | ADRB2 | -0.425023614 | 7 |
| CLC | -0.407493207 | 7 | MBTD1 | -0.425699125 | 7 |
| HIVEP2 | -0.408378921 | 7 | TEF | -0.426286924 | 7 |
| HRH1 | -0.411045656 | 7 | HSD3B2 | -0.427477561 | 7 |
| SART3 | -0.411173712 | 7 | STK10 | -0.428595734 | 7 |
| STUB1 | -0.412103194 | 7 | PNMA3 | -0.430518637 | 7 |
| SMARCC2 | -0.414182127 | 7 | PTGER4 | -0.432836878 | 7 |
| DIDO1 | -0.414744753 | 7 | FCF1 | -0.434379445 | 7 |
| MALT1 | -0.415114677 | 7 | ACVR2A | -0.435212793 | 7 |
| PTPN11 | -0.416696904 | 7 | COBL | -0.437157394 | 7 |
| BUB3 | -0.416955012 | 7 | RPL37A | -0.439515437 | 7 |
| IFT57 | -0.417003359 | 7 | PNMA1 | -0.440541788 | 7 |
| PLOD3 | -0.417062358 | 7 | TCL1A | -0.442043098 | 7 |
| DGKA | -0.417220676 | 7 | RABEP2 | -0.442230421 | 7 |
| ACSBG2 | -0.417291731 | 7 | CDC23 | -0.44720231 | 7 |
| MTCP1 | -0.418160312 | 7 | SMYD2 | -0.447376561 | 7 |
| FAM134B | -0.418595194 | 7 | CYP2R1 | -0.447900759 | 7 |
