# Supplementary figures and images for: Deciphering novel common gene signatures for rheumatoid arthritis and systemic lupus erythematosus by integrative analysis of transcriptomic profiles
Source: PLoS One. 2023 Mar 16;18(3):e0281637. doi: 10.1371/journal.pone.0281637 (PMC10019710; doi:10.1371/journal.pone.0281637)

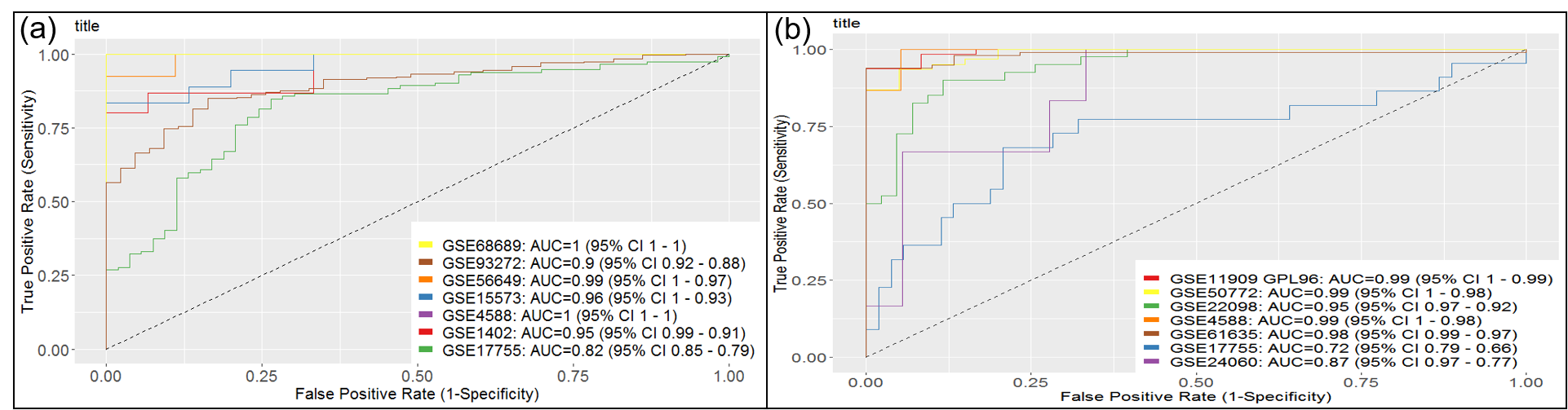

Supplement: S1 Fig — Precision recall Curves for RA (a) and SLE (b). The average precision ranges from the frequency of positive examples ranging from 0.5 (for balanced data) to 1.0 (perfect model). Here, the precision-recall curves represent individual studies from PBMC, WB, and CD4 T and B cell samples. (TIF) [file pone.0281637.s006.tif]

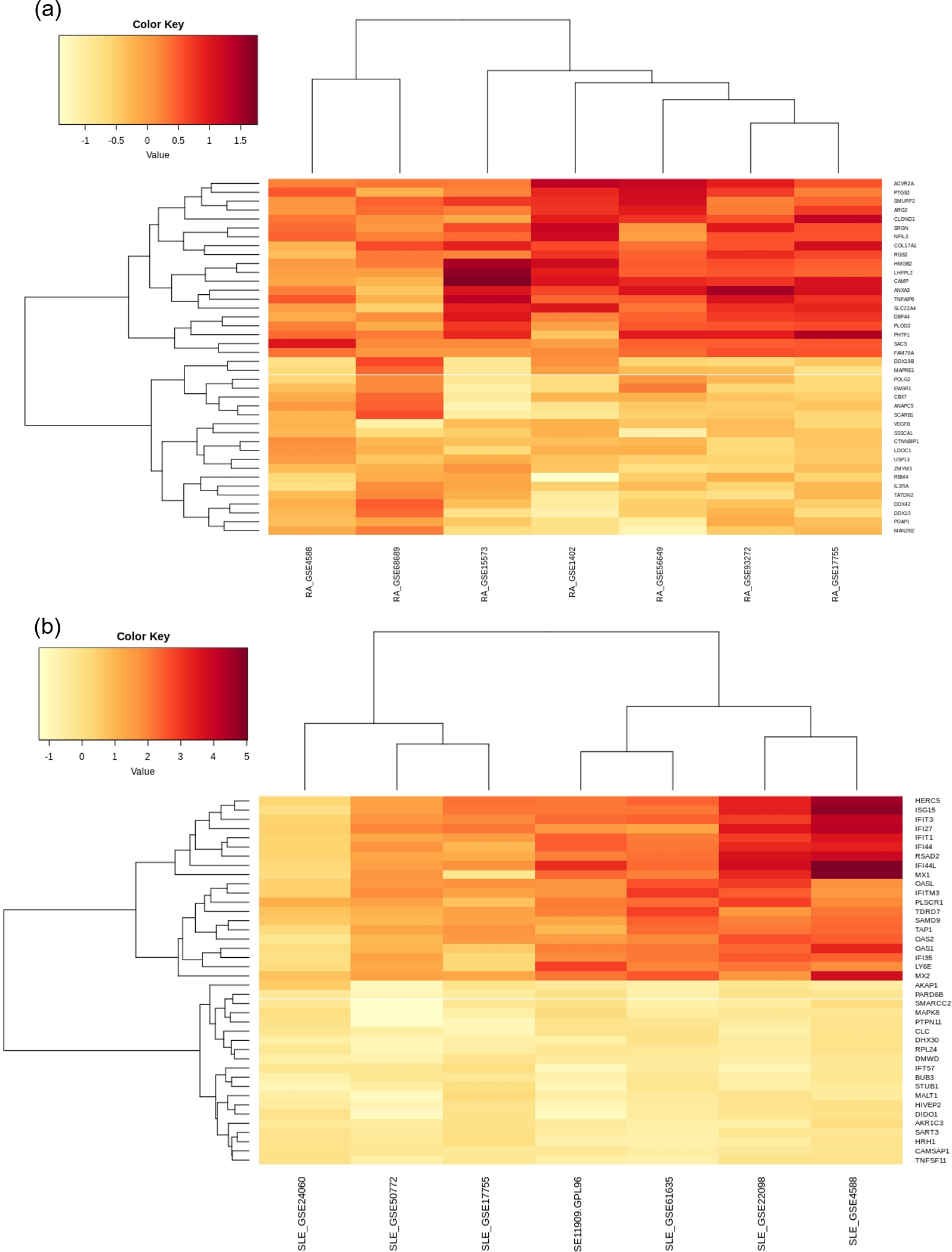

Supplement: S2 Fig — Heatmaps represent the effect size of differentially expressed gene signatures across all datasets (a) top RA DEGs and (b) top SLE DEGs. (Filtering criteria: Effect size > = 0.4 and FDR < = 0.05). Each column is a dataset and each row represents the expression level of the particular gene in all datasets. The colour scale represents the pooled effect size of that particular gene ranging from yellow (low expression) to red (high expression). (TIF) [file pone.0281637.s007.tif]

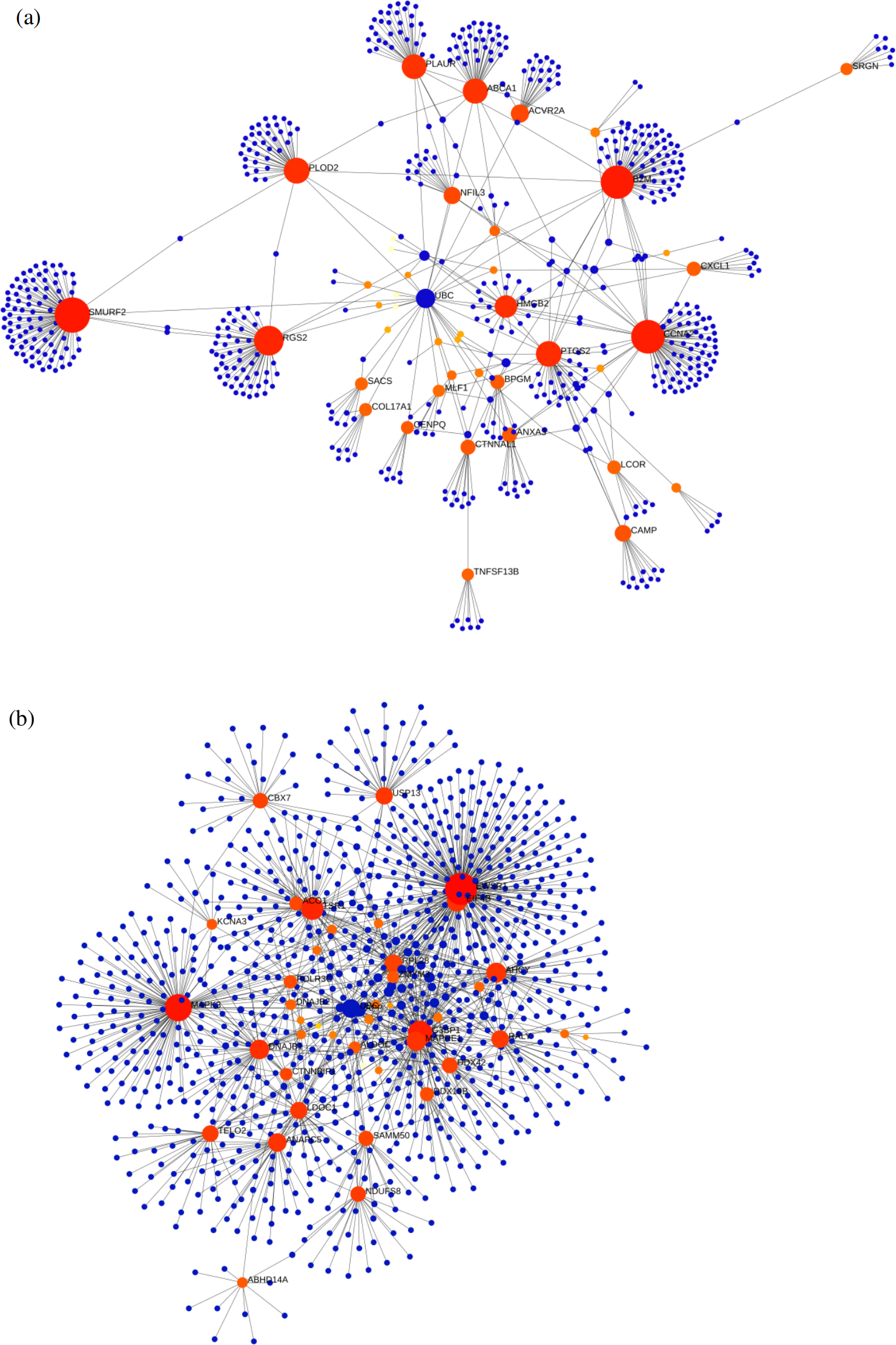

Supplement: S3 Fig — (a) Upregulated RA genes, (b) Downregulated RA genes. The size and the colour of the nodes are layout by the degree and betweenness values. (TIF) [file pone.0281637.s008.tif]

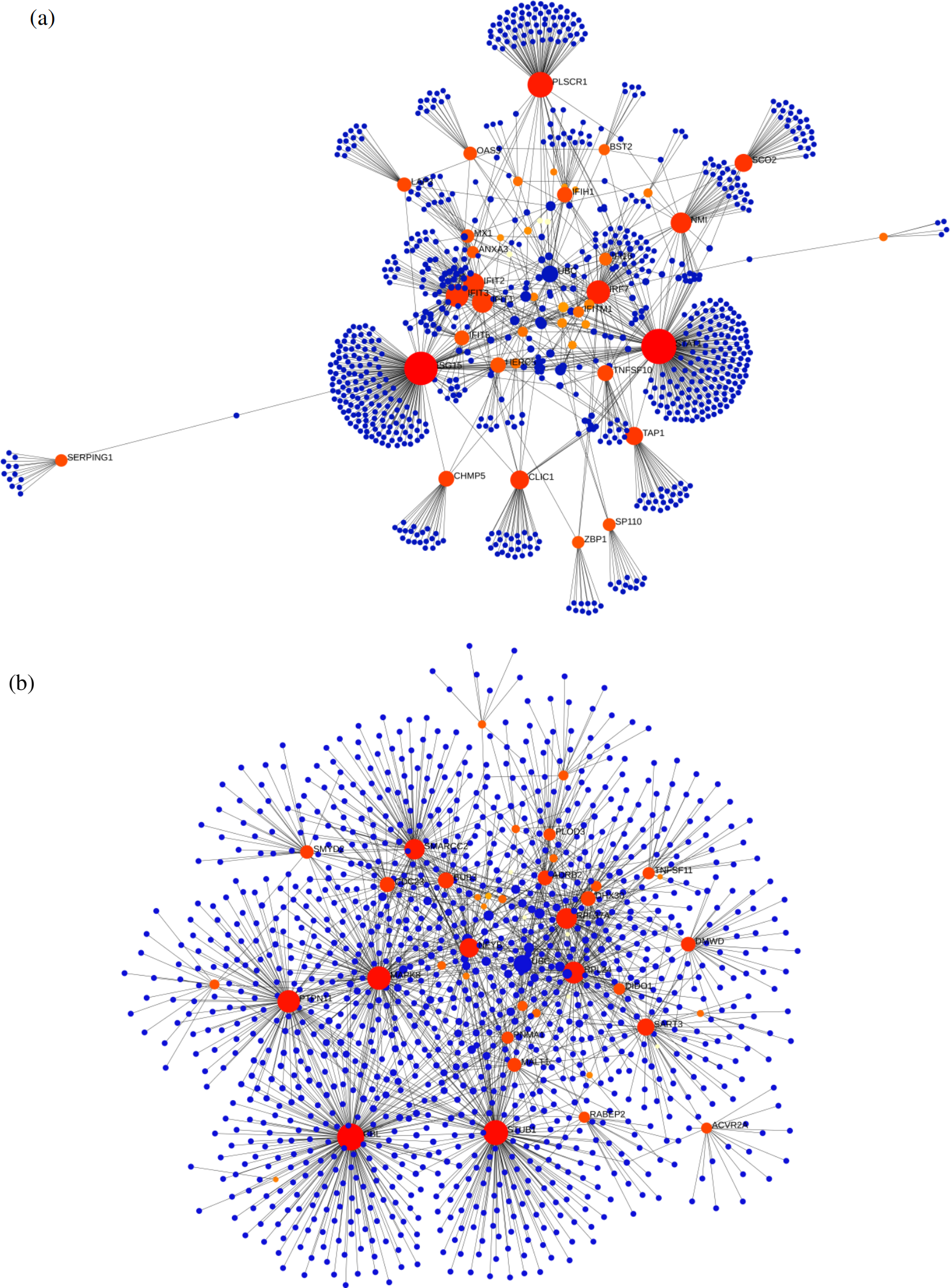

Supplement: S4 Fig — (a) Upregulated SLE genes, and (b) Downregulated SLE genes. The size and the colour of the nodes are layout by the degree and betweenness values. (TIF) [file pone.0281637.s009.tif]
