# Supplementary material for: Deciphering novel common gene signatures for rheumatoid arthritis and systemic lupus erythematosus by integrative analysis of transcriptomic profiles
Source: PLoS One. 2023 Mar 16;18(3):e0281637. doi: 10.1371/journal.pone.0281637 (PMC10019710; doi:10.1371/journal.pone.0281637)
Supplement: Attachment — Submitted filename: Rebuttal_letter_for_plos_one.docx [file pone.0281637.s010.docx]

Dear Editor,

We are thankful for the insightful comments of the reviewers and for providing us an opportunity to revise our manuscript. Our response to the concerns raised by reviewers is given below.

**Q.1.** Manuscript needs to provide limitations of the analysis. Data needs to account for the heterogeneity of the population, disease and the cell population used. Also, reasons for excluding any data set needs to be included. While both RA and lupus are autoimmune diseases, the rationale of discovering common gene expression between the 2 diseases needs to be explained.

**Ans:** Thanks for the comment and suggestion. We have mentioned the limitations of our analysis in the discussion part last paragraph (page no. 23). Meta-analysis allows the integration of multiple heterogeneous datasets to identify robust and reproducible signatures by considering the heterogeneity observed in the real-world patient population. We specifically employed MetaIntegrator for the study as it shortlists robust gene signatures independent of factors such as cell type, genetic background, sex, age, etc., leveraging the biological and technical heterogeneity in these datasets. We have mentioned the exclusion criteria in the results; data pre-processing section (page no. 8).

Autoimmune diseases share several disease mechanisms; as a result, they may have similar characteristics or etiologies. We aimed to provide shared gene signatures related to important pathological functions that can be explored for their therapeutic potential. Although many attempts were made in this direction on many autoimmune diseases, here we are focusing on the most prevalent autoimmune diseases, i.e., RA and SLE. In addition, we have conducted a meta-analysis on sufficient samples to grab the robust common gene signature that underlies the similar pathologic mechanism of the two diseases.

**Reviewer #1:**

**# Major comments**

**Q.1.** Why have only two autoimmune diseases (RA and SLE) been considered to identify the shared gene expression signatures? If the study aimed to identify the shared gene signatures in autoimmune diseases. Wouldn't it be better to consider more autoimmune diseases in the analysis?

**Ans:** There have been several attempts to identify shared gene signatures between various autoimmune diseases, including but not limited to SLE, myositis, RA, scleroderma, Crohn's disease, ulcerative colitis, Sjogren's syndrome (SjS) and type 1 diabetes (T1D). Our study primarily focused on RA and SLE as they are highly prevalent. Even though they are heterogeneous diseases, they manifest similar clinical and pathogenic features, and thus, a differential diagnosis between these immune disorders at an early stage is not always reliable. We appreciate your idea of considering more autoimmune diseases and would certainly include them in our future studies, however it is beyond the scope of the current study.

**Q.2.** As mentioned in the manuscript, a meta-analysis was performed to identify common gene expression signatures for RA and SLE. However, based on the information provided in the Methods section, I don't think the authors have performed a meta-analysis. A meta-analysis, by definition, is a statistical analysis that combines the results of multiple scientific studies addressing the same question.

**Ans:** We have modified the methods section to clarify that the methodology adopted in this paper is indeed a meta-analysis as it combines gene expression data from various studies, all focused on identifying a set of genes to be used as disease signatures for RA and SLE. Metaintegrator achieves that by combining the effect sizes across different datasets from various studies.

**Q.3.** Combining the microarray gene expression profiles of patients (RA and SLE) and healthy controls from the different studies would cause batch effects, which can lead to inaccurate conclusions.

**Ans:** Yes, we strongly agree with the reviewer that combining gene expression profiles from the different studies would cause batch effects and lead to an inaccurate conclusion. However, we have mentioned in the Methods section [Page no. 6, line 111; Para: Data pre-processing and meta-analysis] that MetaIntegrator deals with the problem of batch effects by estimating effect sizes for each dataset independently and then pooling these effect sizes across the datasets supplemented with Cochrane's Q value and a Fisher's p-value to account for heterogeneity.

**Q.4.** In the study, why only 14 gene expression datasets were considered while many more datasets from patients with RA and SLE are available here: https://doi.org/10.1186/s12859-021-04268-4; <https://adex.genyo.es/?>

**Ans:** ADEx collects its data from the GEO only. The datasets described in ADEx were retrieved in the initial steps of our analysis but were excluded at later stages following the inclusion/exclusion criteria (Results section; Data pre-processing; page no. 8). We excluded datasets that were missing healthy controls, including drug treatment, or included gene expression from tissues such as synovial fluid, skin and other cells like monocytes, neutrophils, and other endothelial progenitor cells. The AUROC value indicates how well the MetaScore distinguished between SLE patient samples versus healthy controls. Thus datasets that decreased the summary area under the receiver operating characteristic curve (AUROC) were also excluded.

**Q.5.** L.155–157. Given the fact that there is a vast difference in the number of differentially expressed genes (DEGs) between RA and SLE, only 62 commonly expressed genes between both diseases do not indicate the shared biological mechanism in both autoimmune disorders.

**Ans:** These 62 genes were selected based on a stringent criterion and were present across >4 studies, out of which 30 genes were significantly differentially expressed in all datasets of RA and SLE independent of age, sex, genetic background, and cell type. We focused on these genes as they hold significant importance in the disease biology of RA and SLE. We have also validated the functions of the identified genes from the literature that further ensured their important role in understanding the common pathogenesis mechanism of the two diseases.

**Q.6.** Suppose someone is interested in knowing the shared expressed genes between autoimmune diseases. In that case, they could get that information from Autoimmune Diseases Explorer (https://adex.genyo.es) database. What advantage your study provides over the “Autoimmune Diseases Explorer” database?

**Ans:** The meta-analysis framework used in ADEx uses Rank product for the identification of DEGs, which is different from the one employed in our study. In our study, we used the concept of effect size to predict a subset of genes identified as disease signatures. We also observed many novel genes in our study which were not reported earlier as common gene signatures of RA and SLE. We have used the gene expression meta-analysis tool (MetaIntegartor), which leverages biological and technical heterogeneity to identify a robust disease signature, and has been successful in diverse diseases.

It would not be feasible to draw comparisons between the two as many of the datasets used in our study are not available in ADEx.

\# **Minor comments**

**Q.1.** L.101. What is “individual meta-analysis”?

**Ans:** We have conducted the meta-analysis for both diseases separately by taking seven datasets of RA and 7 for SLE, termed as individual meta-analysis. To avoid further confusion, we have changed it to an independent meta-analysis.

**Q.2.** The quality of the figures is not good, but this could be an issue of the resolution being compromised when generating the review document.

**Ans:** We have rechecked the quality of figures.

**Q.3.** Figure 2 needs to be more precise; it should be remade to see the number of commonly expressed genes between RA and SLE.

**Ans:** We have generated the venn diagram to provide a more detailed view of the commonly expressed genes. It gives detailed information about the 62 genes. The figure clearly shows genes upregulated/downregulated in both diseases and genes with antagonistic expression profiles between RA and SLE. We have explored their roles in the pathogenesis of RA and SLE.

**Reviewer #2**

**# Major comments**

**Q.1.** The study is based upon the meta-analysis of transcriptomic datasets that have been generated on hugely different immune cells populations, i.e. whole blood, PBMCs, CD4 T cells and B cells. Meta-analyses should be performed on homogenous datasets of data, as it is highly misleading to integrate datasets that include a variety of different immune subpopulations.

It would be much more insightful to work on homogenous datasets, and also, possibly, when mixed cell populations are studied, to correct the data for the variable proportions of the different subpopulations (this could be inferred by in silico deconvolution methods)

**Ans:** The reproducibility crisis forms the crux of homogeneous studies where the findings from these studies fail when validated in multiple independent cohorts. Meta-analysis allows the integration of multiple heterogeneous datasets to identify robust and reproducible signatures by considering the heterogeneity observed in the real-world patient population. Several studies have been published by integrating data from WB and PBMCs, excluding other tissues such as synovial fluid, lung tissues etc., to ensure comparable gene expression (Toro-Domínguez D et al., 2014; Wang L et al., 2015; Luan M et al., 2017).

We specifically used MetaIntegrator as it promises robust gene signatures independent of factors such as cell type, genetic background, sex, age, etc., by leveraging these datasets' biological and technical heterogeneity. Results of some of these studies using Metaintegrator have already been further validated in experimental settings (Khatri et al., 2013; Andres-Terre et al., 2015; Sweeney et al., 2016).

**Q.2.** It is not clear why RA and SLE are being compared. The two diseases are characterized by strikingly different pathogenetic processes. What is the final aim of the study? Finding a common expression signature to be used in the diagnosis? or finding pathogenetic processes to be pharmacologically targeted? I would have rather compared more similar diseases, for instance RA, ankylosing spondylitis, psoriatic arthritis, Reiter's syndrome…

**Ans:** Both diseases present clinical heterogeneity, but it has been reported that both RA and SLE share common gene expression signatures in PBMC of all patients with RA and SLE (Maas et al., 2002). Thus, we aimed to compare RA and SLE to find out the genes that are common and related to their pathogenesis using a large number of datasets via a meta-analysis approach. We selected a subset of the identified genes to be robust gene signatures expressed independently of cell type, age, sex, platform, and genetic background.

We aimed to provide shared gene signatures related to important pathologic functions that can be explored for their therapeutic potential.

**References:**

Toro-Domínguez D, Carmona-Sáez P, Alarcón-Riquelme ME. Shared signatures between rheumatoid arthritis, systemic lupus erythematosus and Sjögren’s syndrome uncovered through gene expression meta-analysis. Arthritis Res Ther. 2014;16: 1–8. doi:10.1186/s13075-014-0489-x

Wang L, Wu LF, Lu X, Mo XB, Tang ZX, Lei SF, et al. Integrated analyses of gene expression profiles digs out common markers for rheumatic diseases. PLoS One. 2015;10: 1–11. doi:10.1371/journal.pone.0137522

Luan M, Shang Z, Teng Y, Chen X, Zhang M, Lv H, et al. The shared and specific mechanism of four autoimmune diseases. Oncotarget. 2017;8: 108355–108374. doi:10.18632/oncotarget.19383

Khatri P, Roedder S, Kimura N, De Vusser K, Morgan AA, Gong Y, Fischbein MP, Robbins RC, Naesens M, Butte AJ, Sarwal MM. A common rejection module (CRM) for acute rejection across multiple organs identifies novel therapeutics for organ transplantation. J Exp Med. 2013 Oct 21;210(11):2205-21. doi: 10.1084/jem.20122709.

Andres-Terre M, McGuire HM, Pouliot Y, Bongen E, Sweeney TE, Tato CM, Khatri P. Integrated, Multi-cohort Analysis Identifies Conserved Transcriptional Signatures across Multiple Respiratory Viruses. Immunity. 2015 Dec 15;43(6):1199-211. doi: 10.1016/j.immuni.2015.11.003.

Sweeney TE, Wong HR, Khatri P. Robust classification of bacterial and viral infections via integrated host gene expression diagnostics. Sci Transl Med. 2016 Jul 6;8(346):346ra91. doi: 10.1126/scitranslmed.aaf7165.

Maas, K., Chan, S., Parker, J., Slater, A., Moore, J., Olsen, N. and Aune, T.M. (2002) Cutting edge: molecular portrait of human autoimmune disease. J. Immunol., 169, 5–9.
