# Supplementary material for: Deciphering novel common gene signatures for rheumatoid arthritis and systemic lupus erythematosus by integrative analysis of transcriptomic profiles
Source: PLoS One. 2023 Mar 16;18(3):e0281637. doi: 10.1371/journal.pone.0281637 (PMC10019710; doi:10.1371/journal.pone.0281637)
Supplement: Attachment — Submitted filename: Rebuttal_2_plos_one_final.docx [file pone.0281637.s011.docx]

Dear Editor,

We are thankful for the insightful comments of the reviewers and for providing us an opportunity to revise our manuscript. Our response to the concerns raised by reviewers is given below.

Q.1. The study is based on analysis of various datasets, hence it is recommended that the exclusion criteria for data not included be sound and not based on a bias outcome. The criteria for inclusion and a sound analysis strategy along with the question that is being addressed, pathogenesis vs markers of autoimmunity, needs clarification. The revision needs to address these questions diligently for acceptance. why were certain data using whole blood samples excluded and whether that decision was based on the question being addressed or other reasons? Please define your goals clearly and provide conclusions supported strongly by the analysis.

**Ans:** We have carefully tried to address all the questions raised. We have modified the manuscript to make our objective clear along with reframing the inclusion/exclusion criteria. We have also provided a supplementary table in which we have provided the exclusion details for each dataset. We have changed the conclusion section to better align with our objective.

Additional Editor Comments:

Q.2. For the study to be technically sound, criteria for excluding and inclusion of various datasets needs to be detailed. How the batch effects are dealt in this analysis and the limitations of analysis needs to be explained. Whether the decision to include a dataset was made previous to analysis or excluded later poses a bias and needs to be explained.

**Ans:** We have provided a supplementary table where we have given the details about the exclusion criterion for each dataset. Also, we have made changes to the manuscript for better understanding of the criteria followed.

To maximally overcome the impact caused by the differences in study design and platform usage among different datasets or Batch effects, Combined Effect Size (ES) analysis and Random Effect Modeling (REM) were applied. This eventually results in more consistent and accurate results by taking into consideration both direction and magnitude of gene expression changes.

We have included a paragraph in the Discussion section describing the limitations of our study.

The decision to include/exclude a dataset has been made prior to meta-analysis, hence leaving no question of a biased approach. AUROC is used as a selection criteria to obtain a dataset that holds true discriminatory power to differentiate between healthy and diseased samples. Eventually, this dataset after meta-analysis will yield a set of genes which will serve as robust biomarkers irrespective of the heterogeneity in the real world patient population.

Reviewers' comments:

Reviewer #1: Major comments:
1. As per inclusion criteria, gene expression datasets from whole blood were included in the analysis. However, this needs to be clarified why the following gene expression datasets: GSE45291, GSE61635, GSE65391, GSE72509, and GSE108497, were excluded, while all of them are from whole blood. Also, please provide a supplementary table for excluded datasets mentioning the exclusion criteria based upon that dataset was excluded.

**Ans:** We have used whole blood datasets in this study. The dataset GSE61635 is included in our study. GSE45291 dataset was excluded as this study involved patients receiving drug treatment which is one of the filtering criteria. GSE65391 was filtered based on AUROC. GSE72509 is not a part of our study as this is an RNA-Seq study and we worked with microarray datasets only. GSE108497 was excluded because it involved samples from pregnant women.

As per your suggestion, we have provided a supplementary Table S1 listing each dataset and its exclusion criterion. We have also updated the methods and results sections for the same in the manuscript.

2. Authors responded that "The AUROC value indicates how well the MetaScore distinguished between SLE patient samples versus healthy controls. Thus datasets that decreased the summary area under the receiver operating characteristic curve (AUROC) were also excluded." My reply to authors' response: A decision on exclusion criteria should be made before starting the analysis, but this exclusion criteria seems like a selective exclusion of some datasets that did not work according to the authors' expectations.

**Ans:** The goal of the study was to identify a set of dysregulated genes that are ideal for understanding the molecular mechanisms underlying the pathogenesis of the two most prevalent autoimmune diseases. Thus, in the first stage of filtering, we excluded datasets that involve patients with any drug treatment, unrelated/duplicated tissue samples, and missing healthy controls. The filtering at the second stage based on AUROC was crucial for our proposed objective as it ensured we were using datasets that truly discriminate between healthy vs diseased patients. Eventually, the genes predicted from this data would serve as robust biomarkers irrespective of biological/technical heterogeneity. Additionally, we have also validated the gene signatures identified in this study by taking matched AUROC filtered datasets of RA and SLE from ADEX (https://adex.genyo.es/) database. From our 62 genes list, 30 genes matched the biomarker list generated from mixed tissues ADEX datasets. This further validates the correctness of our approach.

Minor comments:
1. In the Table 1, GSE61635 and GSE24060 are mentioned as from PBMC and whole blood, respectively. However, these should be opposite. GSE61635 from whole blood and GSE24060 from PBMC. Please fix this typo.

**Ans:** Thank you for pointing this out. We have corrected the Table 1 for the same.

Reviewer #2: The authors have not addressed any of the issues raised in the previous round of revision.

Q.1. It is wrong to perform a meta-analysis of datasets generated from different mixed cell populations. Even if the method that the authors have used for the meta-analysis is based on the random model of effect size, this method can only account for small biases among datasets, such as batch effect, but it is not adeguate for integrating data from strikingly different datasets.

**Ans:** We have designed our hypothesis based on previous studies conducted in the same direction to explore the common disease signatures for the two systemic autoimmune diseases on a large sample set. For example, Toro-Domínguez D et al., 2014, conducted a study with objective to perform a meta-analysis using publicly available gene expression data about the three diseases including rheumatoid arthritis, systemic lupus erythematosus and Sjögren’s syndrome to identify shared gene expression signatures. They have taken whole blood, PBMC and CD4 T cell and B cell datasets together for the meta-analysis to investigate the common gene expression signatures shared by the three diseases. Wang L et al., 2015, integrated data from 4 types of rheumatic diseases, and from two different tissue type.

In our study to maximally overcome the impact caused by the differences in study design and platform usage among different datasets, **Combining Effect Size (ES)** analysis and **Random Effect Modeling (REM)** were applied to achieve more consistent and accurate results by taking into consideration both direction and magnitude of gene expression changes. Also In the process of data integration, patient samples from different sources were not segregated, for the purpose to reveal the common gene signature in the two diseases.

We have also validated the gene signatures identified in this study by taking matched AUROC filtered datasets of RA and SLE from ADEX (https://adex.genyo.es/) database. From our 62 genes list, 30 genes matched the biomarker list generated from mixed tissues ADEX datasets. This further validates the correctness of our approach.

Q.2. The authors have not even tried to better define the aims and scopes of this study, and most importantly they have not given explanation on why RA and SLE are being compared. The etiopathogenesis and response to treatment are very different for these two disorders, hence it would be much more insightful to compare more similar diseases, as previously suggested.

**Ans:** As per your suggestion, we have modified our manuscript to better define our end goal.

We compared RA and SLE as the pathophysiology of these two is related in genetic, environmental, and immunological aspects. Previous clinical and epidemiological studies have suggested a shared genetic architecture between these two diseases.

Additionally, several studies have been conducted in this direction in past by Daniel Toro-Domínguez et al., 2014, Icen M et al., 2009, Wang et al., 2022, Lim J 2019. A recent GWAS study revealed common genetic components between RA and SLE and provides candidate-associated loci for the understanding of the molecular mechanism underlying the comorbidity of the two diseases [Lu H et al., 2021]. These previous studies also provided evidence for the shared genetic architecture of RA and SLE. Based on these, we proposed our hypothesis to identify common gene expression patterns, hub genes, commonly regulated important pathways, and regulatory biomarkers involved in the disease mechanism of RA and SLE.

**References:**

Toro-Domínguez, D., Carmona-Sáez, P. & Alarcón-Riquelme, M.E. Shared signatures between rheumatoid arthritis, systemic lupus erythematosus and Sjögren’s syndrome uncovered through gene expression meta-analysis. Arthritis Res Ther 16, 489 (2014). <https://doi.org/10.1186/s13075-014-0489-x.>

Icen M, Nicola PJ, Maradit-Kremers H, Crowson CS, Therneau TM, Matteson EL, Gabriel SE. Systemic lupus erythematosus features in rheumatoid arthritis and their effect on overall mortality. J Rheumatol. 2009 Jan;36(1):50-7. doi: 10.3899/jrheum.080091. PMID: 19004043; PMCID: PMC2836232.

Wang Y, Xie X, Zhang C, Su M, Gao S, Wang J, Lu C, Lin Q, Lin J, Matucci-Cerinic M, Furst DE, Zhang G. Rheumatoid arthritis, systemic lupus erythematosus and primary Sjögren's syndrome shared megakaryocyte expansion in peripheral blood. Ann Rheum Dis. 2022 Mar;81(3):379-385. doi: 10.1136/annrheumdis-2021-220066. Epub 2021 Aug 30. PMID: 34462261; PMCID: PMC8862024.

Lim J, Kim K. Genetic variants differentially associated with rheumatoid arthritis and systemic lupus erythematosus reveal the disease-specific biology. Sci Rep. 2019 Feb 25;9(1):2739. doi: 10.1038/s41598-019-39132-2. PMID: 30804378; PMCID: PMC6390106.

Lu H, Zhang J, Jiang Z, Zhang M, Wang T, Zhao H, Zeng P. Detection of Genetic Overlap Between Rheumatoid Arthritis and Systemic Lupus Erythematosus Using GWAS Summary Statistics. Front Genet. 2021 Mar 18;12:656545. doi: 10.3389/fgene.2021.656545. PMID: 33815486; PMCID: PMC8012913.
